# Cocreating a Climate and Health Dashboard With Multisector Stakeholders: Qualitative Pilot Study

**DOI:** 10.2196/102953

**Published:** 2026-10-29

**Authors:** Cori C Grant, Summer Chavez, Omolola E Adepoju

**Affiliations:** 1 Humana Integrated Health Systems Sciences Institute University of Houston, 5055 Medical Cir, Houston, TX, 77204, United States, 1 713-743-9706; 2 Tilman J Fertitta Family College of Medicine, University of Houston, Houston, TX, United States

**Keywords:** climate and health dashboard, disaster preparedness, digital public health, user-centered design, community engagement, climate resilience, geographic information system, GIS, qualitative research, implementation, disaster response

## Abstract

**Background:**

Climate-related disasters increasingly affect community health, health care access, and disaster preparedness, particularly in disaster-prone Gulf Coast communities. Although climate dashboards have expanded access to environmental data, many function primarily as static repositories with limited integration of health data or operational decision support capabilities.

**Objective:**

This study aimed to identify stakeholder-informed design priorities and implementation considerations for a climate and health dashboard to support disaster preparedness, response, recovery, and community decision-making.

**Methods:**

We conducted semistructured interviews with 7 multisector stakeholders representing academic research, community-based organizations, public health, emergency preparedness, and disaster recovery settings in Houston, Texas. Interviews explored current use of climate and health data, desired dashboard features, usability considerations, data integration needs, and sustainability priorities. Transcripts were analyzed using the Rapid and Rigorous Qualitative Data Analysis (RADaR) technique.

**Results:**

Participants described a need for dashboards that could support decision-making rather than simply display data. Priorities included the integration of climate, health, infrastructure, and social vulnerability data; interactive mapping and layered visualizations; local geographic detail; AI-assisted interpretation and plain language summaries; and tools useful to both community and research audiences. Potential uses included emergency preparedness, targeted outreach during disasters, resource allocation, policy advocacy, and community education.

**Conclusions:**

Findings suggest that effective climate and health dashboards require not only technical functionality but also community-centered design, operational usability, and sustainable implementation infrastructure. These findings are preliminary and may inform future studies aimed at developing dashboards designed to support community-focused disaster preparedness and response.

## Introduction

Gulf Coast communities are disproportionately affected by hurricanes, flooding, and severe storms, which have increased in both frequency and severity in recent years. These disasters contribute to widespread damage to infrastructure and property, disruptions in access to essential medical services, and declines in physical and psychological health [1-3]. These patterns have not only led some to characterize climate-related disasters as a social determinant of health [4,5] but have also highlighted the need for more accessible data, community-engaged planning, and stronger linkages between environmental and health data to support preparedness, response, and recovery efforts [6-9].

In response, numerous climate dashboards and mapping tools have emerged to visualize environmental exposures and associated risks. Although these tools represent important advances in data transparency, many function primarily as static data repositories, with limited integration of health, infrastructure, or social vulnerability data. Existing dashboards frequently rely on fragmented datasets, focus on limited geographic regions, or lack operational functionality capable of supporting actionable decision-making during disasters [10]. Additionally, few studies have examined how multisector stakeholders, including community organizations, emergency preparedness leaders, researchers, and public health practitioners, define the functional and implementation requirements necessary for climate and health dashboards to support real-world preparedness and response.

Human-centered and stakeholder-informed design approaches are increasingly recognized as critical for the development of effective digital public health tools. Understanding how intended users interpret, apply, and operationalize climate and health information may improve dashboard usability, trust, accessibility, and long-term adoption [11-13]. To inform the development of a climate and health dashboard responsive to multisector needs, qualitative interviews were conducted with stakeholders representing community-based organizations, emergency preparedness, public health, disaster recovery, and academic research. This study aimed to identify stakeholder-informed design priorities and implementation considerations for a climate and health dashboard to support disaster preparedness, response, recovery, and community decision-making.

## Methods

### Ethical Considerations

This study was reviewed and approved by the University of Houston Institutional Review Board (STUDY00005665). All participants provided consent prior to participation. Interviews were audio-recorded with participant consent, transcribed verbatim, and analyzed using deidentified data to protect participant confidentiality. All study procedures were conducted in accordance with institutional ethical standards for research involving human participants. Participants were compensated with a US $50 gift card for their participation.

### Study Design

Semistructured interviews were conducted to identify stakeholder priorities for the development of a climate and health dashboard. The study was guided by principles of user-centered and human-centered digital health design.

### Participants and Setting

We used purposive sampling to recruit stakeholders representing key user groups likely to interact with climate and health dashboards. Participants were selected to reflect diverse perspectives across academic research, community-based organizations, public health surveillance, disaster recovery, and policy or advocacy settings. Participants were purposively selected based on their experience and expertise relevant to climate, health, public health surveillance, disaster response, community needs, and policy. These individuals were contacted by email with a brief description of the study and an invitation to participate. Many had previously collaborated with the Humana Institute on related initiatives, providing familiarity with the local context and substantive knowledge relevant to dashboard development. Because the study addressed a narrowly defined research question and recruited information-rich participants representing the intended stakeholder domains, a relatively small sample was considered appropriate for identifying priorities to inform initial dashboard development. Stakeholders identified for recruitment received an email invitation describing the study and inviting them to participate. The sample size was bounded by the formative scope of the study and was designed to capture perspectives across key stakeholder groups rather than to achieve thematic saturation. Saturation was therefore not used as a formal stopping criterion, although recurring priorities were observed across stakeholder interviews.

Participants were based in Houston, Texas, a large urban region with recurrent exposure to hurricanes, flooding, extreme heat, and other climate-related disasters.

### Data Collection

Approximately 60-minute interviews were conducted virtually using Microsoft Teams between December 2025 and February 2026. All interviews were conducted in English.

An interview guide was developed to explore participants’ current use of climate and health data; analytic and decision-making needs; desired dashboard features (eg, visualization, interactivity, data overlays, and data export capabilities); data integration challenges; usability and accessibility considerations; and priorities for dissemination, sustainability, and implementation.

Interviews were conducted by 2 coinvestigators. One had extensive expertise in qualitative research and interviewing, while the second had prior interviewing experience and received additional training from the more experienced coinvestigator.

All interviews were audio-recorded with participant consent and transcribed verbatim using Microsoft Teams. Each interviewer compared the corresponding video recording with the transcript word for word to verify its accuracy, and the transcripts were corrected as needed.

### Data Analysis

Transcripts were analyzed using the Rapid and Rigorous Qualitative Data Analysis (RADaR) technique [14]. Two members of the research team independently reviewed each transcript and imported relevant text into a structured Microsoft Excel matrix organized by participant, topic area, and preliminary analytic notes. Analysis proceeded through iterative phases of data reduction, during which transcripts were systematically condensed by retaining content directly relevant to dashboard functionality, data integration, usability, and implementation considerations.

Text segments were assigned descriptive codes, which were iteratively refined and grouped into categories based on conceptual similarity. Through team-based discussion and consensus, categories were synthesized into overarching themes reflecting stakeholder priorities for dashboard development and implementation. Representative quotes were then selected to illustrate each theme.

### Rigor and Trustworthiness

Members of the research team have expertise in implementation science, public health, climate and health, and community-engaged research, and approached the study with an interest in developing dashboards that are actionable and responsive to stakeholder needs. Recognizing that these perspectives could influence data collection and interpretation, the team used semistructured interviews to encourage participants to describe priorities in their own terms and remained attentive to viewpoints that differed from researchers’ expectations.

Several strategies were used to enhance the rigor and trustworthiness of the analysis. Independent coding by 2 researchers supported credibility and reduced reliance on a single analytic perspective. An audit trail documenting coding decisions, theme refinement, and analytic iterations was maintained throughout the study. Themes were also compared across stakeholder roles to ensure that the findings reflected diverse perspectives and use cases.

## Results

### Overview

A total of 7 participants completed interviews. The sample included 2 academic researchers in environmental health, 1 state emergency preparedness leader, 1 local public health official, and 3 representatives from community-based organizations involved in disaster recovery and advocacy. Participants were classified as representing academic institutions (2/7, 28.6%) and nonprofit organizations (5/7; 71.4% Table 1). Across interviews, participants consistently emphasized that climate and health dashboards should function as operational decision support tools rather than passive data repositories. Participants described dashboards as platforms capable of supporting emergency preparedness, targeted outreach, community education, policy advocacy, and disaster recovery planning (Table 2).

**Table 1.** Participant characteristics (N=7).

Characteristics	Participants, n (%)
Organization type	
Academic or research	2 (28.6)
Community-based or nonprofit	5 (71.4)
Primary role	
Research or data analysis	2 (28.6)
Program or operations	2 (28.6)
Policy or advocacy	3 (42.8)
Primary use of climate and health data	
Research or surveillance	3 (42.8)
Emergency preparedness or planning	1 (14.3)
Community outreach or advocacy	3 (42.8)

**Table 2.** Stakeholder-identified priorities for an actionable climate and health dashboard design.

Themes and representative quote^a^	Dashboard development implication
Data to action: “People don’t want to just see information and not know what to do about it.”	Incorporate AI-generated plain language summaries and actionable guidance
Data integration and granularity for targeted outreach	
“There’s a lot of information... not granular enough... I can’t get to [people].”	Incorporate granular geographic risk stratification capabilities to support targeted outreach during disasters
“We already use FEMA flood layers, but having that aggregated with health data would be good.”	Integrate climate, infrastructure, and health datasets within a unified platform to support operational decision-making
“The dashboard could allow us to overlay... power outages and social vulnerability... to target outreach.”	Enable multilayer geographic information system overlays to identify high-risk populations and prioritize emergency response activities
Interactive design: “Simple tools, like dropdown menus and search functions, would be helpful.”	Design intuitive user interfaces with accessible navigation tools for both technical and nontechnical users
Community co-design	
“It should have community input from conceptualization through testing.”	Incorporate stakeholder engagement throughout dashboard development, testing, and refinement
“If it doesn’t have the community voice... you’re just making a tool for yourself.”	Integrate community co-design processes throughout dashboard development
Policy applications	
“Marginalized communities could demonstrate the need... for making investments.”	Support resource allocation and planning
“This kind of map can be used to guide decisions... where to put a warming center.”	Develop decision support tools to inform infrastructure placement, emergency preparedness, and equity-focused policy decisions
Sustainability	
“Funding to update and make changes... makes it sustainable.”	Plan for long-term maintenance and updates
“You need spokespersons to spread the word... so people can find it.”	Develop dissemination and community communication strategies to support awareness, adoption, and sustained use

a Quotes have been lightly edited for clarity.

### From Data to Actionable Decision Support

Participants highlighted a gap between data availability and actual interpretation, particularly for community users. Stakeholders emphasized that dashboards should translate complex climate and health information into clear, understandable guidance that can support real-world decisions.

One stakeholder noted the following:

There is a gap between providing data and translating it so community members can understand...there is a lot of jargon that needs simple language.

Another stakeholder emphasized as follows:

People don’t want to just see information and not know what to do about it.

Participants recommended incorporating plain language summaries, key takeaways, and AI-enabled interpretation tools to help users understand relationships between variables and emerging risks.

### Dashboard Functionality and Interactive Features

Participants consistently identified interactive visualization tools as essential dashboard features. Stakeholders emphasized the importance of maps, multilayer overlays, scatter plots, search tools, zoom functions, and dynamic visualizations. One participant stated the following:

Visualization features are central to the usefulness of the dashboard.

Another participant noted the following:

It’s easier when it works fast... when you can zoom in and use a lookup tool.

Participants also described the importance of layering climate, infrastructure, and health data to support targeted outreach and emergency response.

### Integrated and Granular Data Infrastructure

Participants emphasized that existing data systems are frequently fragmented and insufficiently granular for operational use. Stakeholders described difficulties in integrating datasets that vary across geographic scales, time frequencies, and data types. One participant explained as follows:

Datasets differ in type, time frequency, and geographic level, which makes them hard to combine.

Another participant described harmonizing data across systems as “a persistent and complex challenge.”

Participants highlighted the need for integrated climate, health, infrastructure, and social vulnerability data within a single platform. Several stakeholders emphasized that granular geographic information is critical for targeted outreach to vulnerable populations during disasters.

### Community Trust and Co-Design

Community engagement emerged as a central implementation priority. Participants emphasized that dashboards are more likely to be trusted and adopted when developed collaboratively with community stakeholders. One stakeholder stated the following:

It should have community input from conceptualization through testing.

Another stakeholder emphasized as follows:

If it doesn’t have the community voice... you’re just making a tool for yourself.

Participants also highlighted the importance of trusted dissemination pathways, including community spokespersons and accessible communication strategies.

### Policy, Equity, and Community Applications

Stakeholders described dashboards as tools capable of supporting equity-focused planning, policy advocacy, and resource allocation decisions. Participants noted that integrated mapping systems could help communities demonstrate infrastructure and service needs. One participant explained as follows:

[Marginalized communities could] demonstrate the need...for making investments.

Another participant described potential uses such as identifying locations for warming centers and other disaster-related infrastructure.

### Sustainability and Future Development

Finally, participants emphasized that sustained funding, technical infrastructure, and ongoing maintenance are necessary for long-term success. Participants cautioned that even well-designed dashboards may fail without continued support for updates, data integration, and dissemination. Stakeholders also identified future opportunities for AI-enabled interpretation, scenario modeling, and educational applications.

## Discussion

### Principal Findings

Findings from this formative study suggest several priorities for the development of climate and health dashboards to support disaster preparedness and response. First, the participants emphasized the value of integrating multiple types of data, including climate exposures, health outcomes, infrastructure disruptions, and social vulnerability indicators. Participants noted that viewing these data together may be more useful for decision-making.

Second, participants described the importance of designing dashboards for different users, including researchers, public health agencies, community organizations, and community members. Suggested features included layered mapping, zoom and search capabilities, data overlays, export capabilities, and simple navigation. Finally, participants identified a need for tools that help users interpret the data, including plain language summaries, key takeaways, and AI-assisted narrative explanations. These features may make dashboard information more accessible to nontechnical users and easier to interpret during disaster events.

Although this is a pilot study, our findings align with established literature on human-centered design, community-based participatory research, and disaster health informatics. Themes related to “community trust and co-design” and “integrated and granular data infrastructure” are consistent with prior research emphasizing that public dashboards need to be easy to navigate for diverse audiences while remaining flexible enough to address different user needs [15,16]. The inclusion of photos and narrative descriptions may also foster a greater sense of connection to and ownership of climate and health dashboards, potentially contributing to trust and sustained use [17,18]. Integrating quantitative data with qualitative and narrative information may therefore make dashboards not only more informative but also more responsive to how communities understand and experience local climate and health risks. Consistent with findings from dashboards implemented in health care quality settings, our findings also highlight the importance of psychological safety and the continuous development of trust among stakeholders [19].

We also intentionally presented nontraditional data sources together, including climate and health data, nonmedical drivers of health, service centers, and place-based characteristics, to illustrate the interconnected effects of extreme weather events, vulnerability, and health. Adding storyboards provides a humanizing element that may be overlooked in other forms of health communication. Other climate and health dashboards, including one using data from the Norwegian Quality of Life Survey, have similarly demonstrated the potential value of combining infographics with dashboards to strengthen user engagement [20]. These considerations may be particularly important in disaster health informatics, where preparedness and response depend on translating complex environmental and health data into locally meaningful information that communities can use to identify vulnerabilities, prioritize resources, and strengthen resilience [18]. Collectively, these approaches may help to position the climate dashboard not merely as an information resource but as an evolving tool that communities and organizations can use to understand local conditions, advocate for resources, and support action.

### Limitations

This study has several limitations. First, the sample size was small and geographically concentrated in Houston, Texas, which may limit transferability to rural or noncoastal settings. Second, participants primarily represented organizational stakeholders with prior experience using climate and health data, which may not fully reflect the perspectives of broader community populations or individuals with limited digital literacy. Finally, because the dashboard had not yet been fully developed or deployed, participants were responding to conceptual features and anticipated use cases rather than interacting directly with a functioning platform. Despite these limitations, the study provides important formative insights into stakeholder priorities for the development of climate and health dashboards to support disaster preparedness and public health decision-making.

### Conclusions

Findings from this pilot study suggest that dashboards may have value beyond passive visualization by serving as preparedness and operational decision support tools that inform action during disasters. Human-centered and community-informed design approaches may help enhance the usability, trust, and adoption of these tools among communities. Future research should build on these findings through prototype development, usability testing, iterative refinement, and evaluation of implementation within real-world disaster preparedness and response workflows.

## Abbreviations

RADaR: Rapid and Rigorous Qualitative Data Analysis
